# Lungenseparation im Kindesalter: Narkoseführung mit physiologischen und technischen Herausforderungen

**DOI:** 10.1007/s00101-025-01599-2

**Published:** 2025-10-21

**Authors:** Christoph Geier, Christiane E. Beck, Jan Karsten, Katja Nickel

**Affiliations:** Klinik für Anästhesiologie und Intensivmedizin, Medizinische Hochschule Hannover, Carl-Neuberg-Str. 1, 30625 Hannover, Deutschland

**Keywords:** Kinderanästhesie, Pädiatrisches Atemwegsmanagement, Thoraxchirurgie, Einlungenventilation, Bronchusblocker, Doppellumentubus, Pediatric anesthesia, Pediatric airway management, Thoracic surgery, Single-lung ventilation, Bronchial blocker, Double-lumen tube

## Abstract

Eine Lungenseparation für thorakale Eingriffe ist in Abhängigkeit von der Altersgruppe mit unterschiedlichen Schwierigkeiten verbunden. Während die anatomischen, physiologischen und technischen Voraussetzungen bei einer entsprechenden Auswahl an Bronchusblockern und Doppellumentuben bei Kindern > 12 Jahren vergleichbar zu Erwachsenen sind, gilt dies für Neugeborene und Säuglinge nicht. Sie bilden aber mit 13 % die zweitgrößte Gruppe von Kindern, die einen lungenchirurgischen Eingriff benötigen. Eine Lungenseparation in dieser Altersgruppe ist technisch anspruchsvoll. Die Rate für kardiopulmonale Komplikationen ist um ein Vielfaches höher als bei älteren Kindern und Erwachsenen. Diese Eingriffe werden dadurch zu einer anästhesiologischen Herausforderung, die nur in kinderanästhesiologischen Zentren mit einer entsprechenden personellen und strukturellen Ausstattung erfolgen sollten. Auch bei Säuglingen sollte eine Extubation *on table* angestrebt werden, um die Erholungszeit zu verkürzen. Voraussetzung dafür ist eine suffiziente und multimodale Schmerztherapie. Für eine qualitativ hochwertige perioperative Behandlung kann die Entwicklung eines abteilungsinternen ERAS-Protokolls hilfreich sein.

**Zusatzmaterial online:**

Die Online-Version dieses Artikels (10.1007/s00101-025-01599-2) enthält das PDF der Kitteltaschenkarte zur Lungenseparation im Kindesalter. Bitte scannen Sie den QR-Code.

## Abstract (en)

Lung separation for thoracic surgery is associated with varying degrees of difficulty depending on the age group. While the anatomical, physiological and technical requirements for the appropriate selection of bronchial blockers and double-lumen tubes in children > 12 years of age are comparable to those for adults, this is not the case for newborns and infants; however, at 13% they constitute the second largest group of children requiring lung surgery. Lung separation in this age group is technically demanding. The rate of cardiopulmonary complications is many times higher than in older children and adults. These procedures are therefore an anesthesiological challenge that should only be performed in specialized pediatric anesthesiology centers with appropriate personnel and structural facilities. In infants extubation should also be attempted on table to shorten the recovery time. This requires sufficient and multimodal pain therapy. The development of an internal enhanced recovery after surgery (ERAS) protocol can be helpful for high-quality perioperative treatment.

Die Einlungenventilation (ELV) im Kindesalter stellt eine seltene anästhesiologische Technik, die nur an wenigen Zentren in Deutschland praktiziert und gelehrt wird, dar. Viele anästhesiologisch tätige Ärztinnen und Ärzte haben nur selten die Gelegenheit, diese durchzuführen und entsprechende Routine zu entwickeln [1]. In diesem Beitrag wird auf pathophysiologische Besonderheiten, unterschiedliche Techniken der ELV in Abhängigkeit vom Patientenalter sowie auf das Ziel der Enhanced Recovery after Surgery (ERAS) eingegangen.

Die ELV hat sich in den vergangenen Jahren kontinuierlich weiterentwickelt. Sie kann zur Erleichterung der chirurgischen Darstellung sowie zur anatomischen Isolierung einer Lungenseite z. B. bei Blutungen, Infektionen, Fisteln oder im Rahmen einer Ganzlungenwaschung bei alveolärer Proteinose verwendet werden. Mögliche chirurgische Eingriffe, bei denen sie im Kindesalter zum Einsatz kommt, sind pulmonale Eingriffe wie die Versorgung kongenitaler Malformationen der Lunge, Lungenteilresektionen sowie pleurale und extrapleurale Pathologien. Auch für extrapulmonale Eingriffe, beispielsweise in der thorakalen Gefäßchirurgie, Ösophaguschirurgie oder Wirbelsäulenchirurgie, kann eine ELV notwendig sein [2].

Eine Auswertung von Krankenversicherungsdaten ergab, dass pädiatrische lungenchirurgische Eingriffe vorwiegend in den Altersgruppen < 1 Jahr (13 %) bzw. > 14 Jahre (50 %) durchgeführt wurden. In der Gruppe der Kinder < 1 Jahr wurden überwiegend kongenitale Lungenmalformationen versorgt. Der Begriff kongenitale Lungenmalformation wird als Oberbegriff für eine Vielzahl von Erkrankungen verwendet. Dazu zählen die kongenitale pulmonale Atemwegsmalformation („congenital pulmonary airway malformation“, CPAM), die intra- und extralobäre Lungensequestration, bronchogene Zysten, angeborene große hyperluzente Lappen („congenital large hyperlucent lobe“, CLHL) im Rahmen eines kongenitalen lobären Emphysems („congenital lobar emphysema“, CLE) sowie die Bronchialatresie.

Bei den über 14-jährigen werden vorwiegend Pneumothoraces operativ behandelt [3]. Grundsätzlich gibt es im Kindesalter verschiedene Ansätze zur ELV: die Platzierung eines Bronchusblockers, eines Doppellumentubus (DLT) oder die endobronchiale Intubation. Die technischen Möglichkeiten hierfür haben sich in den vergangenen Jahren deutlich verbessert, was v. a. für die Versorgung sehr kleiner Kinder (< 1 Jahr) neue operative Möglichkeiten eröffnet.

Dieser Artikel gibt einen Überblick über aktuelle Entwicklungen und deren praktische Implikationen bei der noch immer seltenen Durchführung der ELV im Kindesalter.

## Pathophysiologische Bedingungen bei Kindern

Bei wachen spontan atmenden Kindern in sitzender oder stehender Position ist das Verhältnis aus Ventilation und Perfusion (V/Q-Verhältnis) über die ganze Lunge (fast) ausgeglichen. Die Ventilation nimmt in kraniokaudaler Richtung zu; die Perfusion nimmt (aufgrund des hydrostatischen Gradienten) ebenfalls zu. Faktoren wie Allgemeinanästhesie ohne Spontanatmung, ELV oder Seitenlagerung beeinflussen das V/Q-Verhältnis negativ.

Kinder (insbesondere kleine Kinder) sind von den Folgen der ELV stärker betroffen als Erwachsene, da sich die anatomischen und physiologischen Voraussetzungen, einem Shunt entgegenzuwirken, im Laufe der Entwicklung vom Kind bis zum Jugendlichen erst ausbilden (Veränderungen des Knorpel/Knochen-Verhältnisses des Thorax, Größenzunahme und, damit verbunden, größerer Einfluss des hydrostatischen Gradienten, Metabolismus = Verhältnis von Sauerstoffangebot und -verbrauch im Verlauf der Entwicklung vom Kind zum Erwachsenen).

**Neugeborene** und **Säuglinge** haben eine (absolut) geringere Zahl an Alveolen. Dies führt zu einer geringeren Lungen-Compliance. Diese Altersgruppe weist auch eine geringere Muskelspannung der Brustwand und konsekutiv eine geringere nach außen gerichtete Kraft der Thoraxwand auf [4, 5]. Dies schafft ein Ungleichgewicht gegenüber den nach innen gerichteten elastischen Rückstellkräften der Lungen (*elastic recoil*): Die funktionelle Residualkapazität (FRC) ist reduziert. Der Mangel an Muskeltonus in der Brustwand führt dazu, dass das alveoläre Verschlussvolumen (*closing volume,* CV) höher als die FRC ist und in der Folge zu einer erhöhten Kollapsneigung der Atemwege (*airway closure*) und der Ausbildung von Atelektasen führt (Gefahr der Hypoxämie). Weniger Alveolen, eine geringere FRC und kleinere Atemwegsdurchmesser tragen außerdem zu einem höheren Atemwegswiderstand in dieser Altersgruppe bei.

Die Lungenphysiologie ab dem Kleinkindalter zeichnet sich durch eine deutlich größere Zahl an Alveolen aus, die sich auch in die Adoleszenz hinein weiter vermehren, was mit einer Abnahme des Atemwegswiderstandes und einer Zunahme der Lungen-Compliance einhergeht. Mit 2 Jahren ermöglicht eine zunehmende Brustwandmuskulatur und eine Ossifikation der Rippen eine ausreichende *Gegenkraft* zum *Elastic recoil* der Lunge. Die Folgen der (geringeren) FRC-Abnahme können deutlich weniger beobachtet werden [6].

Diese unterschiedlichen Entwicklungsstadien bedingen auch, ob man eine ELV durchführen kann oder nicht. Viele Studien zeigen, dass eine ELV bei Kindern und Jugendlichen für thorakoskopische und offene Eingriffe möglich und die Komplikationsrate dabei gering ist [6–9]. In der klinischen Praxis bleibt die Durchführung der ELV im Hinblick auf die pathophysiologischen Konsequenzen eine individuelle Herausforderung [4, 9, 10].

## Pathophysiologie in Seitenlage

Wir gehen davon aus, dass die Kinder für die meisten thoraxchirurgischen Operationen in Seitenlage liegen, und sprechen daher von einer abhängigen (unten liegenden) Lunge und einer nichtabhängigen (oben liegenden) Lunge.

Die Perfusion der Lunge ist in Seitenlage schwerkraft-abhängig. Geht man davon aus, dass zu Beginn der Operation in Seitenlage noch beide Lungen beatmet werden, so beträgt der pulmonale Blutfluss (PBF) durch das hydrostatische Gefälle in der oberen, nichtabhängigen Lunge ca. 40 % und in der unteren, abhängigen Lunge ca. 60 %. Durch die geringere Körpergröße von Kindern ist dieser Mechanismus weniger ausgeprägt als bei Erwachsenen. In der Seitenlagerung kommt es zur Ausbildung von (Kompressions‑)Atelektasen durch den intraabdominellen Druck, das Mediastinum und die geringere Compliance der Brustwand. Diese Kombination aus Zunahme der Perfusion und regionaler Ventilationsstörung führt zu einem intrapulmonalen Shunt (Mismatch aus Ventilation und Perfusion), der klinisch in aller Regel unbedeutend ist.

## Pathophysiologie bei Einlungenventilation

Durch die ELV wird die nichtabhängige Lunge nicht mehr belüftet, aber perfundiert. Dadurch entsteht ein jetzt deutliches Ventilations‑/Perfusions-Mismatch (V/Q-Mismatch; [4, 11]).

Die Totalatelektase der Lunge bei ELV und die damit verbundene Abnahme der Compliance führt zu einer Umverteilung der regionalen Ventilation in die noch beatmete Lunge. Der alveoläre Sauerstoffpartialdruck (p_A_O_2_) fällt ab. Diese alveoläre Hypoxie ist ein Trigger für die hypoxisch-pulmonale Vasokonstriktion (HPV, Euler-Liljestrand-Mechanismus). Die HPV ist ein autoregulativer Reflexmechanismus, der den PBF umverteilt und den pulmonalen Shunt reduziert. Im Gegensatz zur „Zweilungenventilation“ fließen nur noch ca. 20 % des pulmonalen Blutflusses durch die nichtabhängige Lunge, während bis zu 80 % durch die abhängige Lunge fließen [4]. Die erste Reflexantwort erfolgt nach wenigen Sekunden mit einem Peak nach 15–20 min, wobei es eine zweite Reflexantwort nach 1–2 h gibt.

Bei Kindern sind die Kompensationsmechanismen im Vergleich zu Erwachsenen beschränkter und werden jetzt in Form einer Hypoxämie deutlich. Durch ihre geringere Körpergröße ist das hydrostatische Gefälle zur abhängigen Lunge geringer bei gleichzeitig eingeschränkter Compliance und Ventilation der abhängigen Lunge.

Die HPV wird durch folgende Faktoren vermindert:

- Hypotension,
- Hypothermie,
- Hypokapnie,
- hohe Tidalvolumina,
- exzessiv hoher PEEP,
- Alkalose,
- volatile Anästhetika (dosisabhängiger Effekt),
- Inflammation,
- exzessive Flüssigkeitszufuhr.

Stickstoffmonoxid (NO) vermindert potent den Mechanismus der HPV. Die Applikation von NO bei ELV kann das Shunt-Volumen in der abhängigen Lunge verringern, in dem es lokal die Vasokonstriktion in belüfteten Arealen weiter senkt. Im Rahmen von ELV während einer Lungentransplantationen oder speziellen Indikationen kann es mit diesem Wirkprinzip eingesetzt werden [12–14].

### Keypoints.

- Je kleiner das Kind ist, desto höher ist das Risiko von Hypoxämien während der ELV durch ein V/Q-Mismatch, bedingt durch:

1. Die Kompression der abhängigen Lunge bei weniger starrem knorpeligen Brustkorb und des durch das Zwerchfell übertragenen Bauchdrucks, wodurch Compliance und Ventilation in der abhängigen Lunge verringert werden.
2. Einen geringeren hydrostatischen Gradienten von der nichtabhängigen zur abhängigen Lunge aufgrund der geringeren Größe des Kindes zu einer weniger effektiven Umleitung des Blutes von der nichtabhängigen Lunge zur abhängigen Lunge.

## Allgemeine Techniken der Einlungenventilation

Die unterschiedlichen Techniken der ELV sind abhängig vom Alter und der dadurch sich ergebenden Tubusgröße, die nach der Formel von Motoyama (Alter /4 + 3,5 mm ID (mit Cuff)) berechnet werden kann [15]. Die Tubusgröße bestimmt darüber, ob eine extraluminale oder intraluminale Platzierung eines Blockers möglich ist, wann ein DLT passen kann, und welche Größe Bronchoskope haben können. Tab. 3 zeigt die Größen und möglichen Kombinationen auf.

## Endobronchiale Intubation

Eine ELV kann durch eine endobronchiale Intubation in Abhängigkeit vom Alter mit einem ungecufften oder gecufften Tubus versucht werden [5, 16, 17]. Bei Frühgeborenen kann sie im Rahmen einer Nutzen-Risiko-Abwägung die einzige Möglichkeit der Lungenseparation darstellen.

Der linke Hauptbronchus ist in der Regel um eine halbe Tubusgröße kleiner als der rechte Hauptbronchus. Kinder müssen bei diesem Vorgehen oral intubiert werden, da sonst der Tubus zu kurz ist. Die Platzierung ist durch unterschiedliche Abgangswinkel der Hauptbronchi rechts einfacher als links.

Als kleinster für ein Neugeborenes zur Verfügung stehender Tubus hat der 2,5 Innendurchmesser (ID) ohne Cuff einen Außendurchmesser (AD) von 3,3 mm und kann zur Intubation des linken Hauptbronchus (3,6 mm Ø) genutzt werden. Für den rechten Hauptbronchus mit einem Ø von 4,4 mm kann ein Tubus der Größe 3,0 ohne Block (AD 4,0 mm) verwendet werden. Beachtet werden sollte, dass der Tubusaußendurchmesser abhängig vom Hersteller variiert [2].

**Abb. 1:**
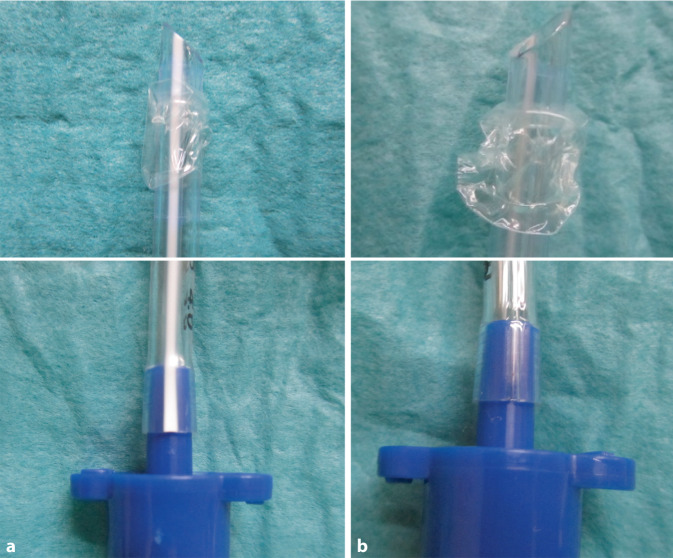
**a** Tubusstreifen oben bedeutet Tubusspitze links, **b** Tubusstreifen unten bedeutet Tubusspitze rechts

Die optimale Platzierung rechts ist anspruchsvoll aufgrund des sehr frühen Abgangs des Oberlappens. Bei einer endobronchialen Intubation ist es hilfreich, für eine Platzierung nach rechts die Tubusspitze bei der Intubation auf der rechten Seite zu haben (Tubusstreifen unten). Bei Tuben mit Murphy-Auge sollte anschließend mit einer bronchoskopischen Lagekontrolle überprüft werden, ob der rechte Oberlappen (OL) über das Murphy-Auge belüftet ist. Tuben ohne Murphy-Auge sollten nach ihrer Platzierung um 180° gedreht werden (Tubusstreifen oben), um eine Belüftung des OL zu ermöglichen (Abb. 1).

**Abb. 2:**
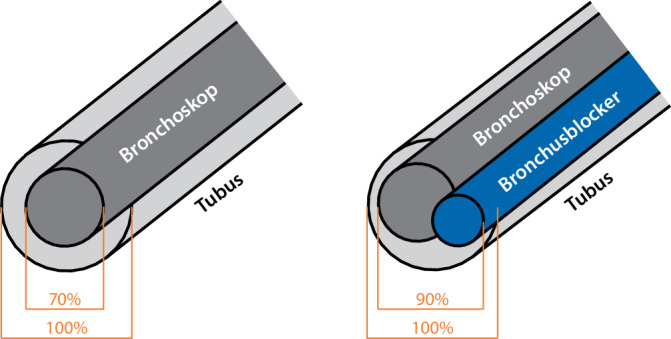
Verhältnisse der Innendurchmesser von Bronchoskop, Bronchusblocker und Endotrachealtubus

Zur Platzierung in den linken Bronchus wird die linke Schulter unterpolstert, der Kopf nach rechts gedreht, und die Tubusspitze liegt während der Intubation links (Tubusstreifen oben). Die bronchoskopische Führung gestaltet sich meist schwierig, da der Tubus rigider ist als ein Bronchoskop mit 1,8 mm AD. Eine Lagekontrolle muss ebenfalls erfolgen.

Der intraoperativ feste Sitz ist ein Vorteil. Ein schneller Wechsel zwischen ELV und Zweilungenventilation (ZLV) ist nicht möglich. Die abgehängte Lunge kollabiert meist unvollständig. Die Absaugung bzw. die Möglichkeit für eine apnoische Oxygenierung der zu operierenden Seite ist nicht möglich ([16]; Tab. 1).

**Tab. 1:** Vor- und Nachteile der einseitigen Intubation

Vorteile	Nachteile
– Platzierung rechts einfach – Relativ fester Sitz intraoperativ – Auffädelung auf Bronchoskop zur Platzierung möglich	– Platzierung links häufig schwieriger – Rechts: Risiko Abhängen des rechten OL – Kein schneller Wechsel zwischen ELV und ZLV – Bei ungecufften Tuben Risiko von Blut- oder Sekretübertritt – Absaugung nicht möglich – Kollaps der operierten Lunge ggf. unvollständig – Apnoische Ventilation der operierten Seite nicht möglich

*OL* Oberlappen, *ELV* Einlungenventilation, *ZLV* Zweilungenventilation

## Einlungenventilation mit Bronchusblocker

Vor dem 8. Lebensjahr ist eine Lungenisolation neben der endobronchialen Intubation in der Regel nur durch einen Bronchusblocker möglich. In der aktuellen Literatur wird beschrieben, dass Bronchusblocker (Arndt-Blocker) auch schon bei Neugeborenen und sehr kleinen Säuglingen genutzt werden [2, 4, 18]. Sie sind in 5 French (F), 7F oder 9F verfügbar und abhängig von der Tubusgröße extraluminal oder intraluminal platzierbar. Die Platzierung kann blind oder mit Auffädelung auf ein Bronchoskop erfolgen. Dafür befindet sich im Blockerkanal eine Schlaufe, mit deren Hilfe der Arndt-Blocker am Bronchoskop fixiert werden kann. Die Schlaufe kann anschließend entfernt werden und die Deflation der Lunge erfolgen; diese kann durch vorsichtiges Absaugen unterstützt werden. Das Vorgehen ist nicht immer erfolgreich. Die anschließende bronchoskopische Lagekontrolle ist obligat.

Alternativ kann der Arndt-Blocker mit einem passenden Gefäßdraht stabilisiert und vorgebogen werden, um dadurch eine Platzierung nach links zu erleichtern [18].

Über den Blocker-Kanal ist eine apnoische Oxygenierung der abgehängten Lunge bei der ELV möglich.

In einer retrospektiven multizentrischen Untersuchung gibt es Hinweise darauf, dass die Nutzung eines Bronchusblockers im Vergleich zur endobronchialen Intubation mit einem geringeren Risiko einer Hypoxämie bei ELV einhergeht [19].

Der Bronchusblocker kann extraluminal neben dem Tubus oder intraluminal im Tubus platziert werden. Die Entscheidung ist abhängig von der Größe des Tubus (ID in Millimeter (mm)), des Bronchoskops (AD in mm) und des Bronchusblockers (AD in F 1F ≙ 0,33 mm). Der Tubus sollte um 0,5 mm kleiner als die errechnete Größe sein, wenn der Bronchusblocker zusätzlich durch die Glottis gelegt wird.

Bei der extraluminalen Platzierung muss nur das Bronchoskop durch den Tubus passen. Für eine Ventilation während der Bronchoskopie sollte der Bronchoskop-AD maximal 70 % des Tubus-ID betragen (Abb. 2). Das kleinste Bronchoskop mit einer Absaugung AD 2,8 mm passt durch einen Tubus ID 3,5 (2,8/3,5 = 0,8). Eine Beatmung ist in diesem Fall ab einem Tubus ID 4,0 möglich (2,8/4,0 = 0,7). Müssen kleinere Tuben verwendet werden, stehen Bronchoskope bis 1,8 mm AD ohne Absaugung zu Verfügung. Aufgrund der fehlenden Absaugmöglichkeit kommt es jedoch mitunter nur zu sehr eingeschränkten Sichtverhältnissen.

Vor der Intubation sollte geprüft werden, ob das Bronchoskop durch den ausgewählten Tubus passt [4]. Bei der Intubation wird der Bronchusblocker vor dem Tubus platziert und anschließend bronchoskopisch die Lage kontrolliert und ggf. korrigiert. Sinnvoll ist es, den Bronchusblocker etwas tiefer zu platzieren und erst in Seitenlage endgültig zu positionieren.

Bei der intraluminalen Platzierung sollte die Summe der AD von Bronchoskop und Bronchusblocker 90 % des ID betragen, eine Ventilation ist in diesem Fall immer möglich, da durch die runden Formen von Bronchoskop und Bronchusblocker oberhalb und unterhalb ausreichend Platz bleibt (Abb. 2).

Das Kind wird intubiert, der Bronchusblocker anschließend über einen im Set befindlichen Adapter platziert und über diesen die Lage auch ohne Unterbrechung der Ventilation bronchoskopisch kontrolliert. Die Platzierung rechts ist durch den steilen Abgang des rechten Hauptbronchus meist unproblematisch. Durch den abgewinkelten Abgang des linken Hauptbronchus ist die extraluminale Positionierung dort häufig schwieriger. Durch das Vorbiegen des Bronchusblockers um 35–45° oberhalb des Cuffs, das Anheben der linken Schulter und eine Kopfdrehung nach rechts wird die Platzierung erleichtert [20].

Das Bronchoskop verursacht fast immer einen Anstieg des Atemwegswiderstandes und eine Abnahme der Ventilation, was bei respiratorisch eingeschränkten Kindern schnell zu einer Dekompensation führen kann.

## Besonderheiten bei Neugeborenen

In der Literatur wird der Einsatz des Arndt-Blockers 5F auch bei Neugeborenen beschrieben [4]. Die Länge des Cuffs vom Arndt-Blocker kann die Nutzung in dieser Altersgruppe aus Sicht der Autoren einschränken. Alternativ kann die Blockung mit einem Fogarty-Katheter 3F oder 4F bzw. einem einlumigen Pulmonaliskatheter 4F in Einzelfällen als Off-Label-Use erwogen werden. Die Katheter werden aufgrund der kleinen Tubusgrößen immer extraluminal zum Tubus platziert. Beide Katheter verfügen über einen niedrigvolumigen Hochdruckballon. Die Position und das Aufblasen des Ballonkatheters müssen bronchoskopisch beobachtet werden, um eine Schädigung der Bronchialschleimhaut zu vermeiden. Schon kleine Volumen- und Druckanpassungen bei diesen Kathetern haben einen erheblichen Einfluss auf die Okklusion und Isolation der Lunge.

Vorteil dieses Verfahrens gegenüber der einseitigen Intubation ist ein Wechsel von ELV auf ZLV zu jeder Zeit (Tab. 2). Damit kann die ELV auch während der Operation kurzzeitig aufgehoben werden, um z. B. zum Ende Verletzungen der Lunge zu suchen und ggf. zu übernähen. Zudem können auch selektiv tiefere Lungenkompartimente abgehängt werden, während die oberen an der Ventilation beteiligt sind. Beides unterstützt eine stabile Oxygenierung während der ELV. Bei der Blockierung der rechten Lunge wird häufig der rechte Oberlappen weiterhin belüftet, da dieser direkt nach der Aufzweigung der Hauptbronchien abgeht. Wenn der Bronchusblocker nur knapp im rechten Hauptbronchus liegt, kann eine Dislokation während der Operation zur Verlegung des gesamten Atemwegs mit Sättigungsabfällen führen. Durch die Entblockung ist eine schnelle Ventilation wieder möglich. Die erneute Platzierung führt häufig zu einer Unterbrechung der Operation. Eine apnoische Ventilation oder die Deflation der abgehängten Lunge ist beim Fogarty-Katheter nicht möglich, da der Katheter nicht kanalisiert ist. Die Entlüftung der Lunge erfolgt durch Absorptionsatelektasen über einen längeren Zeitraum. Das wird durch die Ventilation mit einer F_I_O_2_ von 1,0 unterstützt.

**Tab. 2:** Vor- und Nachteile des Arndt-Blockers

Vorteil	Nachteil
– Jederzeit Wechsel von ELV auf ZLV – Apnoische Oxygenierung über abgehängte Lunge möglich – Auffädelung auf Bronchoskop möglich	– Dislokationsgefahr – Eingeschränkte Deflation der abgehängten Lunge möglich – Rechts: Risiko OL weiterhin belüftet – Links: extraluminal schwieriger zu platzieren

*ELV* Einlungenventilation, *ZLV* Zweilungenventilation, *OL* Oberlappen

## Einlungenventilation mit einem Doppellumentubus

Ab einem Alter von 8 Jahren kann die ELV durch einen DLT erwogen werden. In einer Untersuchung von Seefelder et al. 2012 zeigte sich, dass Kinder, die 2 der folgenden 3 Kriterien erfüllten, mit einem DLT intubiert werden können. Die Kriterien sind: Kinder, die älter als 8 Jahre sind, größer als 130 cm sind oder schwerer als 30 kg sind [21].

Sinnvoll ist es, den Tubus-AD der errechneten Tubusgröße zu ermitteln; dieser steht meist auf der Verpackung. Die Größe des DLT wird als AD in French angegeben (1F = 0,33 mm). Der kleinste verfügbare DLT hat 26F ≙ 8,6 mm AD. Das entspricht einem Einlumentubus (ELT) von 6,5 ID und bedeutet, rechnerisch nach der Formel von Motoyama ist ein DLT ab 12 Jahren denkbar und wurde auch so beschrieben [22]. Aufgrund der Variabilität der Atemwegsgröße und der Vorteile des DLT kann der Einsatz früher erwogen werden. Da DLT sehr rigide sind und eine Verletzungsgefahr besteht, sollte vorher die Intubation mit einem ELT 6,5 ID erfolgreich gewesen sein. Die Autoren haben bei einer Auswertung von Ganzlungenwaschungen über 10 Jahre den DLT bei einem Altersdurchschnitt von 10,3 Jahren genutzt [23]. Vorteile eines DLT sind die sichere Platzierung des Tubus sowie die unkomplizierte Deflation und Absaugung der zu operierende Lunge. Alternativ zu der Intubierbarkeit mit einem Tubus 6,5 ID können der Durchmesser der Trachea (TD) und des linken Hauptbronchus (DLB) über eine Thoraxröntgen- oder CT-/MRT-Bildgebung ausgemessen sowie ein entsprechender DLT gewählt werden. Bei bekanntem TD kann der DLB auch nach einer von Brodsky publizierten Formel berechnet werden [24, 25]: DLT = (0,4 × TD) + 3,3.

Tab. 3 listet die zur Verfügung stehenden Materialen mit Größenangaben und einer Alterszuordnung auf.

**Tab. 3:** Altersbezogene Größen von Tubus und Bronchoskop mit entsprechendem Separationstool und Technik

Alter (Jahre)	Tubus (ID)	Bronchoskop (AD)	Bronchusblocker/DLT	Technik
Neugeborene	3,0 (mit Cuff)	Bis 1,8 mm	Fogarty-Katheter 3F, 4F Pulmonaliskatheter 4F (Arndt-Blocker 5F)	Extraluminal
1.–2. Lj.	3,5 (mit Cuff)	Bis 2,2 mm	Arndt-Blocker 5F (Fogarty-Katheter 4F)	Extraluminal
3.–4. Lj.	4,0 (mit Cuff)	Bis 2,8 mm	Arndt-Blocker 5F	Extraluminal
5.–6. Lj.	4,5–5,0 (mit Cuff)	Bis 3,8 mm	Arndt-Blocker 5F	Intraluminal
7.–8. Lj.	5,0 (mit Cuff)			
9. Lj.	5,5 (mit Cuff)	Bis 4,2 mm	Arndt-Blocker 7F	Intraluminal
10.–12. Lj.	6,0–6,5 (mit Cuff)	Arndt-Blocker bis 4,2 mm DLT bis 2,2 mm	Tubus ID 6,0: Arndt-Blocker 7F Tubus ID 6,5: Wechsel auf DLT 26F	Intraluminal
Ab ca. 13. Lj.	6,5–7,0 (mit Cuff)	DLT bis 2,2 mm	DLT 26F–28F	–
Ab ca. 15. Lj.	7,0 (mit Cuff)	DLT 28F bis 2,2 mm DLT 32F bis 2,8 mm	28F–32F–(35F)	–

*DLT* Doppellumentubus, *ID* Innendurchmesser, *AD* Außendurchmesser, *Lj.* Lebensjahr

Unabhängig von der Technik der ELV sollten das Atemzugvolumen auf < 6 ml/kgKG reduziert und die Atemfrequenz entsprechend erhöht werden [26–28].

Kinder profitieren, wenn die Compliance der abhängigen Lunge durch ein Rekrutierungsverfahren vor Beginn der ELV optimiert wird [29].

### Keypoints.

- Ein DLT kann ab einem Alter von 12 Jahren sicher genutzt werden.
- Ab einem Alter von 8 Jahren kann ein DLT erwogen werden, wenn die Intubation mit einem ELT 6,5 ID erfolgreich gewesen ist.
- Verletzungen aufgrund der Rigidität des DLT müssen unbedingt vermieden werden,

## Narkoseführung mit dem Ziel *Enhanced Recovery after Surgery*

Enhanced-Recovery-after-Surgery(ERAS)-Programme sind ein multidisziplinärer Ansatz mit dem Ziel, die Qualität der perioperativen Versorgung zu steigern und die postoperative Erholung zu beschleunigen. In einer aktuellen Publikation konnte gezeigt werden, dass ERAS-Programme in Single-Center-Studien den Krankenhausaufenthalt verkürzen und das Outcome von Kindern verbessern. [30]. Im Jahr 2020 wurde mit den *Consensus Guidelines for Perioperative Care in Neonatal Intestinal Surgery *durch die ERAS Society die bisher einzige Empfehlung im Bereich der Kinderchirurgie veröffentlicht ([31]; Tab. 4).

**Tab. 4:** Eckpfeiler des anästhesiologischen Managements im Sinne eines ERAS-Protokolls in der pädiatrischen-Thoraxanästhesie

	Ziele	Vorgehen
Intraoperative Homöostase	Normotension Normovolämie Normofrequenz Normokapnie Normoxämie Normothermie Normoglykämie Normonatriämie	Kurze Nüchternzeiten Stündliche BGA (arteriell, zentralvenös) Differenzierte Volumen und Kreislauftherapie PONV-Prophylaxe
Narkoseführung	Wenig Arbeitsplatzbelastung Frühextubation	TIVA mit gut steuerbaren Medikamenten
Narkosemonitoring	Ausreichender MAD, HZV, Endorganperfusion, Gasaustausch	Basis-Monitoring Invasive Blutdruckmessung Säuglinge: NIRS, ZVK (großzügige Indikation)
Schmerztherapie	No Pain Gestufter, multimodaler Ansatz	Nichtopioidanalgetikum Dexamethason Opioid Regionalanästhesie/Lokalanästhesie Thorakoskopie: Wundinfiltration Laterale Thorakotomie: Erector Spinae Plane Block, chirurgische Interkostal‑/Paravertebralblockade tPDK für Jugendliche Sternotomie: Parasternalblock bds. tPDK für Jugendliche, falls keine Regionalanästhesie möglich: Dexmedetomidinperfusor i.v. Lidocainperfusor i.v.

*PONV* Postoperative Nausea and Vomiting, *TIVA* Total Intravenous Anaesthesia, *tPDK* thorakaler Periduralkatheter, *MAD* Mittlerer arterieller Druck, *HZV* Herzzeitvolumen, *BGA* Blutgasanalyse, *NIRS* Nahinfrarotspektroskopie, *ZVK* Zentraler Venenkatheter, *i.v.* intravenös, *bds.* beidseits

Für die Sicherheit und Qualität in der Kinderanästhesie wurden von der Initiative „Safetots.org“ die 10-N-Regeln für Kinderanästhesiologie entwickelt. Sie bestimmen die Pfeiler einer sicheren Narkoseführung und legen den grundlegenden Qualitätsanspruch an eine Kindernarkose fest [32]. Neben diesem Qualitätsanspruch und den im vorangegangenen Kapitel beschriebenen technischen Herausforderungen des Atemwegsmanagements liegt bei Lungeneingriffen der anästhesiologische Fokus auf dem erweiterten hämodynamischen Monitoring, der Beatmung, dem Kreislauf- und Volumenmanagement, einer multimodalen Schmerztherapie und einer frühen Extubation.

Angeborene Malformationen der Lunge, die im Neugeborenenalter operiert werden müssen, gehen mit einer deutlichen Kompromittierung des Kindes einher und bestimmen dadurch den frühen Operationszeitpunkt. Diese Kinder gehen sehr eingeschränkt in die Narkose. Die kleinen anatomischen Verhältnisse führen dazu, dass nach der Lagerung und dem Abdecken des Operationsgebietes kaum noch ein Zugang durch die Anästhesiologie an das Kind besteht. Neben der Notwendigkeit einer invasiven Blutdruckmessung für eine Überwachung des Gasaustausches und des arteriellen Blutdrucks unter der ELV sollte auch die Indikation für einen zentralen Venenkatheter (ZVK) großzügig gestellt werden, damit eine zielgerichtete Kreislauftherapie durch Volumen- und Katecholamingaben gesteuert werden kann. Die Überwachung der regionalen zerebralen Sättigung durch Nahinfrarotspektroskopie sollte bei allen kleinen Kindern unbedingt angewendet werden. Eine operationsbedingte Einschränkung des Herzeitvolumens (HZV) oder eine unbeabsichtigte Hyperventilation bei wechselnden Beatmungssituationen kann dadurch frühzeitig sichtbar werden; dies erhöht die Sicherheit des Kindes.

Die Beatmung muss bei kleinen Kindern häufig an die Operationssituation angepasst werden und kann mit der Notwendigkeit eines Wechsels aus manueller Beatmung und druckkontrollierter Beatmung einhergehen. Die wechselnden intrathorakalen Druckverhältnisse und die kleinen Tidalvolumina in Kombination mit oberen Druckgrenzen bringen moderne Beatmungsgeräte an ihre Grenzen der Kompensation. Wenig tracheobronchiales Sekret verursacht bei enger Anatomie schnell hohe Beatmungsdrücke, die zum Abbruch der Ventilation führen. Mit einer manuellen Flatterbeatmung können diese punktuellen Probleme gelöst werden. Eine permissive Hyperkapnie wird von Kindern in aller Regel problemlos toleriert.

Eine totale intravenöse Anästhesie bietet sich in allen Altersgruppen an, weil sie nicht zu einer Narkosegasbelastung des Personals im Rahmen der notwendigen Bronchoskopien führt und die Narkosezufuhr bei eingeschränkter Ventilation nicht beeinträchtigt ist. Sinnvoll ist der Einsatz kurz wirksamer, gut steuerbarer Medikamente, die eine rasche Extubation *on table* oder zeitnah auf der Intensivstation ermöglichen.

Die Schmerztherapie sollte in allen Altersgruppen einem gestuften, multimodalen Ansatz folgen und ein Regionalanästhesieverfahren, angepasst an den chirurgischen Zugang, enthalten. Die *European Society for Paediatric Anaesthesiology (ESPA) Pain Management Ladder Initiative *erstellte dazu eine sehr detaillierte Handlungsempfehlung für thorakoskopische oder offenen Operationen, inklusive Dosierungen [33]. Während bei Jugendlichen ein thorakaler Periduralkatheter (tPDK) gut anzulegen ist und für den postoperativen Verlauf viel Erfahrung existiert, bringen neuroaxiale Verfahren und Katheteranlagen bei kleinen Kindern Einschränkungen mit sich. Die Schmerzkatheter müssen in Narkose angelegt werden und haben bei mobilen kleinen Kindern oft eine kürzere Liegedauer als gewünscht. Zusätzlich stören sich Kinder häufig an jedem Zugang, Katheter und Pflaster. Die Erfahrung mit den Dosierungen und der Wirkung von Regionalanästhesien bei Kindern ist allgemein niedriger. Dies führt in aller Regel zu der Notwendigkeit einer Monitorüberwachung, die bei eingeschränkten Ressourcen knapp ist. Aus diesem Grund ist ein Faszienblock, wie der Erector Spinae Plane Block, eine geeignete Alternative für alle Altersgruppen. Er ermöglicht eine lang anhaltende Analgesie und eine Reduktion des postoperativen Opioidbedarfs mit einer Single-Shot-Technik. Dadurch kann auf einen Schmerzkatheter verzichtet werden. Der Block kann nach der Operation am noch in Seitenlagerung befindlichen Kind mit wenig Aufwand, zwingend sonographisch gesteuert und an die Schnittführung angepasst, gestochen werden. Es handelt sich dabei um ein der speziellen Kinderanästhesie vorbehaltenes Regionalverfahren [33–36]**.**

Die Schmerztherapie sollte eine rasche Mobilisation auf den Arm oder aus dem Bett ermöglichen und zum Wohlbefinden der Kinder beitragen. Bei Säuglingen ist dafür eine rasche natürliche Nahrungsaufnahme notwendig. Wichtig ist ein guter Übergang der intraoperativ begonnenen in die sich anschließende postoperative Schmerztherapie. Die Schnittstellen zwischen OP-Intensivstation/Aufwachraum und Normalstation müssen durch gemeinsame Schmerzkonzepte und Verantwortlichkeiten geregelt sein. Eine Begleitung dieser Kinder durch den Akutschmerzdienst ist sinnvoll. Kinder > 2 Jahre sollten im Rahmen der ERAS-Strategie eine differenzierte Prophylaxe und Therapie von postoperativer Übelkeit und Erbrechen erhalten, um eine schnelle Erholung und das Wohlbefinden zu fördern [37].

Während sich das anästhesiologische Management von Jugendlichen mit einer Körpergröße und einem Körpergewicht nahe dem von Erwachsenen nicht grundsätzlich von der Behandlung Erwachsener unterscheidet, trifft dies für Neugeborene, Säuglinge und Kleinkinder nicht zu. Diese Gruppe hat ein deutlich erhöhtes Risiko für intraoperative kardiopulmonale Komplikationen. Ein Eingriff an den Atemwegen steigert das Risiko von Komplikationen zusätzlich [38, 39]. Diese Kinder sollten nur in einem Zentrum mit der entsprechenden apparativen und personellen Ausstattung behandelt werden. Dazu gehören neben einer großen Erfahrung in spezieller Kinderanästhesiologie ein multiprofessionelles Team zur Behandlung von Komplikationen und der postoperativen Betreuung.

### Keypoints.

- Das Risiko kardiopulmonaler Komplikationen ist bei Neugeborenen und Säuglingen erhöht. Thoraxeingriffe sollten in diesem Alter nur in spezialisierten kinderanästhesiologischen Zentren durchgeführt werden.
- Ziel: Enhanced Recovery after Surgery durch: multimodale Schmerztherapie und eine frühe Extubation, auch bei Säuglingen

​

**Abb. 3:**
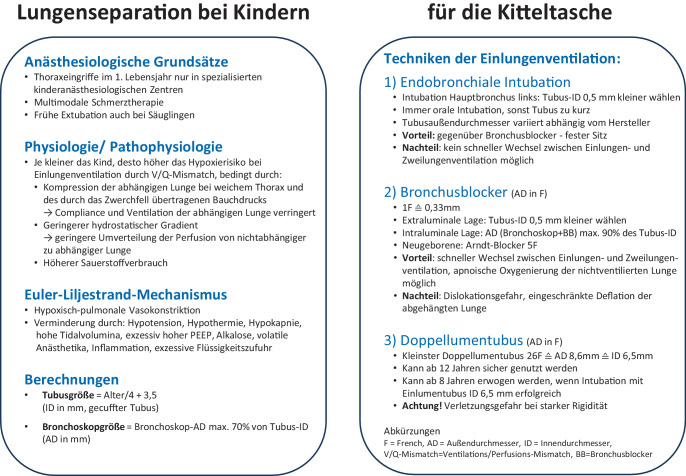
Kitteltaschenkarte zur Lungenseparation im Kindesalter. Ein PDF zum Download finden Sie im Zusatzmaterial des Artikels online

## Fazit für die Praxis

- Mehr als 10 % der Lungeneingriffe finden im ersten Lebensjahr statt.
- Je kleiner das Kind, desto höher ist das Risiko von Hypoxämien während einer Einlungenventilation (ELV).
- Es besteht bei der ELV kleiner Kinder ein V/Q-Mismatch: bedingt durch den noch weichen Thorax mit geringerer Compliance und eingeschränkter Ventilation der abhängigen Lunge sowie einem geringeren hydrostatischen Gradienten von der nichtabhängigen zur abhängigen Lunge aufgrund der geringeren Größe des Kindes.
- Die Verwendung eines Bronchusblockers hat ein geringeres Hypoxämierisiko als die einseitige endobronchiale Intubation und ist ab dem Neugeborenenalter möglich.
- Ein Doppellumentubus (DLT) kann ab einem Alter von 8 Jahren erwogen und ab einem Alter von 12 Jahren sicher genutzt werden.
- Für die sichere Intubation mit dem kleinsten DLT (26 F) sollte die Intubation mit einem ELT 6,5 ID erfolgreich gewesen sein.
- Aufgrund des erhöhten kardiopulmonalen Risikos und des hohen technischen Anspruchs an die Narkose sollten Thoraxeingriffe bei Neugeborenen und Säuglingen nur in spezialisierten kinderanästhesiologischen Zentren erfolgen. Das Ziel ist eine sichere und qualitativ hochwertige Behandlung in allen Altersgruppen, die eine schnelle postoperative Erholung ermöglicht.

## Supplementary Information

Kitteltaschenkarte zur Lungenseparation im Kindesalter
