# Supplementary material for: Lung separation in childhood: Anaesthesia management with physiological and technical challenges
Source: Anaesthesiologie. 2025 Oct 21;74(11):717–26. [Article in German] doi: 10.1007/s00101-025-01599-2 (PMC12552392; doi:10.1007/s00101-025-01599-2)
Supplement: Supplementary file 1 — Kitteltaschenkarte zur Lungenseparation im Kindesalter [file 101_2025_1599_MOESM1_ESM.pdf]

Infokarte für die Kitteltasche: Lungenseparation im Kindesalter

Ausschneiden, falten, gegebenenfalls laminieren und immer informiert sein.

Lungenseparation bei Kindern

hier falten

Anästhesiologische Grundsätze

- Thoraxeingriffe im 1. Lebensjahr nur in spezialisierten kinderanästhesiologischen Zentren
- Multimodale Schmerztherapie
- Frühe Extubation auch bei Säuglingen

Physiologie/ Pathophysiologie

- Je kleiner das Kind, desto höher das Hypoxierisiko bei Einlungenventilation durch V/Q-Mismatch, bedingt durch:
  - Kompression der abhängigen Lunge bei weichem Thorax und des durch das Zwerchfell übertragenen Bauchdrucks → Compliance und Ventilation der abhängigen Lunge verringert
  - Geringerer hydrostatischer Gradient → geringere Umverteilung der Perfusion von nichtabhängiger zu abhängiger Lunge
  - Höherer Sauerstoffverbrauch

hier falten

Euler-Liljestrand-Mechanismus

- Hypoxisch-pulmonale Vasokonstriktion
- Verminderung durch: Hypotension, Hypothermie, Hypokapnie, hohe Tidalvolumina, exzessiv hoher PEEP, Alkalose, volatile Anästhetika, Inflammation, exzessive Flüssigkeitszufuhr

Berechnungen

- **Tubusgröße** = Alter/4 + 3,5 (ID in mm, gecuffter Tubus)
- **Bronchoskopgröße =** Bronchoskop-AD max. 70% von Tubus-ID (AD in mm)

für die Kitteltasche

Techniken der Einlungenventilation:

1) Endobronchiale Intubation

- Intubation Hauptbronchus links: Tubus-ID 0,5mm kleiner wählen
- Immer orale Intubation, sonst Tubus zu kurz
- Tubusaußendurchmesser variiert abhängig vom Hersteller
- **Vorteil** gegenüber Bronchusblocker: Fester Sitz
- **Nachteil** : kein schneller Wechsel zwischen Einlungen- und Zweilungenventilation möglich

2) Bronchusblocker (AD in F)

- 1F ≙ 0,33mm
- Extraluminale Lage: Tubus-ID 0,5mm kleiner wählen
- Intraluminale Lage: AD (Bronchoskop + Bronchusblocker) < 90% Tubus-ID
- Neugeborene: Arndt-Blocker 5F
- **Vorteil**: schneller Wechsel zwischen Einlungen- und Zweilungenventilation, apnoische Oxygenierung der nichtventilierten Lunge möglich
- **Nachteil**: Dislokationsgefahr, eingeschränkte Deflation der abgehängten Lunge

3) Doppellumentubus (AD in F)

- Kleinster Doppellumentubus 26F ≙ AD 8,6mm ≙ ID 6,5mm
- Kann ab 12 Jahren sicher genutzt werden
- Kann ab 8 Jahren erwogen werden, wenn Intubation mit Einlumentubus ID 6,5mm erfolgreich
- **Achtung!** Verletzungsgefahr bei starker Rigidität

Abkürzungen

AD = Außendurchmesser, ID = Innendurchmesser,
F = French, V/Q-Mismatch = Ventilations-Perfusions-Mismatch,

Dieses Angebot ist ein Zusatzmaterial zum Beitrag „**Lungenseparation im Kindesalter“** von Christoph Geier, Christiane E. Beck, Jan Karsten und Katja Nickel (2025) in *Die Anaesthesiologie*

Beitrag und Zusatzmaterial stehen Ihnen auf www.springermedizin.de zur Verfügung. Bitte geben Sie dort den Beitragstitel in die Suche ein.

Springer Medizin
